# The Finite Pore Volume GAB Adsorption Isotherm Model as a Simple Tool to Estimate a Diameter of Cylindrical Nanopores

**DOI:** 10.3390/molecules26061509

**Published:** 2021-03-10

**Authors:** Sylwester Furmaniak, Piotr A. Gauden, Maria Leżańska, Radosław Miśkiewicz, Anna Błajet-Kosicka, Piotr Kowalczyk

**Affiliations:** 1 Stanisław Staszic State University of Applied Sciences in Piła, Podchorążych Street 10, 64-920 Piła, Poland; ablajet@puss.pila.pl; 2 Carbon Materials Application in Electrochemistry and Environmental Protection Research Group, Faculty of Chemistry, Nicolaus Copernicus University in Toruń, Gagarin Street 7, 87-100 Toruń, Poland; gaudi@umk.pl; 3 Research Group for Modeling and Synthesis of Novel Materials, Faculty of Chemistry, Nicolaus Copernicus University in Toruń, Gagarin Street 7, 87-100 Toruń, Poland; miriam10@umk.pl; 4 Faculty of Economics and Management, University of Szczecin, Aleja Papieża Jana Pawła II 22A, 70-453 Szczecin, Poland; Radoslaw.Miskiewicz@usz.edu.pl; 5 School of Engineering and Information Technology, Murdoch University, Murdoch, WA 6150, Australia; P.Kowalczyk@murdoch.edu.au

**Keywords:** cylindrical pores, N_2_ adsorption, isotherm models, type IV isotherm

## Abstract

The finite pore volume Guggenheim–Anderson–de Boer (fpv-GAB) adsorption isotherm model has been considered as a simple tool which not only enables us to analyze the shape of isotherms theoretically, but also provides information about pore diameter. The proposed methodology is based on the geometrical considerations and the division of the adsorption space into two parts: the monolayer and the multilayer space. The ratio of the volumes of these two spaces is unambiguously related to the pore diameter. This ratio can be simply determined from the N_2_ adsorption isotherm by its fitting with the use of fpv-GAB model. The volume ratio is equal to the ratio of the adsorption capacities in the monolayer and the multilayer—two of the best-fit parameters. The suggested approach has been verified using a series of isotherms simulated inside ideal carbon nanotubes. The adsorption data for some real adsorbents has also been used during tests. The studies performed have proven that diameters estimated with the use of the proposed method are comparable with the geometrical sizes or diameters published by others and based on the application of more sophisticated methods. For pores wider than 3 nm, the relative error does not exceed a few percent. The approach based on the fpv-GAB model reflects well the differences in pore sizes for the series of materials. Therefore, it can be treated as a convenient tool to compare various samples.

## 1. Introduction

Nanoporous materials are still of wide interest. This interest results from their many applications, which include, among others, water filters [1], energy and gas storage [2,3], medical implants and drug delivery [4,5,6,7], and catalysis [8]. Nanoporous media are also often used in the chemical industry and in broadly understood nanotechnology [9,10]. The main factor determining properties of such materials is their porous structure, i.e., the geometry and the size of pores and the distribution of these parameters, since the porosity is very often heterogeneous. Among the materials of various pore geometry—which may also be irregular—one can distinguish those with cylindrical pores. This inhomogeneous group includes carbon nanotubes [11,12], some ordered carbons [13] and mineral adsorbents, like MCM-41 [14], AlPO_4_-5 [15], SBA-15 [16], and alumina [17], as other nanoporous materials, also those ones with cylindrical pores, have various applications—see for example [12,18,19,20,21,22,23,24,25,26,27].

The direct determination of a nanopore’s size and its distribution is usually a very difficult task, especially in the case of disordered and amorphous materials with heterogeneous pores. Therefore, the porosity is usually characterized indirectly. The most popular methods used for this purpose are based on the measurements of the low-temperature adsorption isotherms of inert gases—mainly N_2_ [28,29]. Such approaches allow us to determine the pore size distribution (PSD) curve with the use of the global adsorption isotherm equation and the base of the local isotherms for pores of well-defined geometry differing in size [30,31]. The local isotherms are generated using more or less sophisticated mathematical models or from the results of molecular simulations. The fitting of an experimental isotherm by the theoretical one, leading to the PSD, also requires the use of a complicated mathematical and/or computational methodology. However, in practice, it is often very convenient to use a single parameter (single numerical value) instead of the full PSD curve to compare or to characterize the materials. Such a parameter is usually the average size or the size of the dominant pores. These values can often be determined using simpler methods.

Previously, we proposed a mathematical model to describe adsorption isotherms in cylindrical pores that leads to the estimation of the pore diameter during fitting of the data [32]. The formalism of this approach, the so-called nanotube polymolecular Fowler–Guggenheim adsorption isotherm (NT-PFG), is complicated since the adsorption space is divided into layers of molecular thickness. As a consequence, it is required to model the adsorption amounts separately for each subsequent layer. Moreover, from the adsorption mechanism standpoint, the exact distinction of the subsequent layers, with the exception of the monolayer, and several following layers, is problematic. This is because the borders between them become more and more blurred as the distance from the pore wall increases [33,34]. However, the reliability of the predictions of this model were successfully verified for experimental and simulated data [32]. In the current study, we have suggested a simplified approach based on the division of the cylindrical space into two parts only: the monolayer and the multilayer. The ratio of the volumes of these two spaces is unambiguously related to the pore diameter and can be used to calculate it. This value can be estimated from the adsorption isotherm as the ratio of adsorption capacities in the monolayer and the multilayer. We have proposed the use of a theoretical adsorption isotherm model to determine these adsorption capacities. We have applied the finite pore volume Guggenheim–Anderson–de Boer model (fpv-GAB) [35] as one of the few models that allow us to fit type IV adsorption isotherms according to the IUPAC classification and are based on the assumption of the realistic mechanism of the adsorption process. Such an approach allows the use of an isotherm description by the theoretical model to estimate the pore diameter. This is quite convenient since the adsorption data are often analyzed by fitting with the use of different theoretical equations of isotherms. The reliability of the proposed methodology has been verified using the series of N_2_ adsorption isotherms simulated inside carbon nanotubes (CNTs) of different diameters and the experimentally measured data for selected real materials of the cylindrical pores.

## 2. Materials and Methods

### 2.1. Mathematical Formalism of Adsorption Modeling

**Figure 1:**
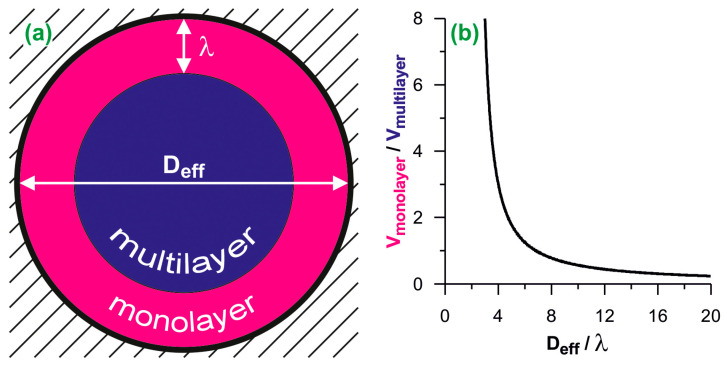
(**a**) The scheme of the division of cylindrical adsorption space into the monolayer and the multilayer. *D_eff_* is the effective diameter and λ is the monolayer thickness; (**b**) the ratio of monolayer and multilayer volumes plotted as the function of the effective diameter. The diameters are reduced by the monolayer thickness.

We have based our analysis on the assumption that the space available for adsorption inside a cylindrical pore can be unambiguously divided into two parts: that occupied by the monolayer (contact layer) of the adsorbed fluid, and the central part, where the multilayer adsorption is observed (see Figure 1a). The geometric ratio of volumes of these parts (*x*) is equal to:

(1) $$x=\frac{V_{monolayer}}{V_{multilayer}}=\frac{D_{eff}^{2}-{\left(D_{eff}-2\lambda \right)}^{2}}{{\left(D_{eff}-2\lambda \right)}^{2}}$$

where *D_eff_* is the effective pore diameter and *λ* is the monolayer thickness. For N_2_ used as the adsorbate in the present work we have assumed *λ* = 0.35 nm [36]. Since the ratio *x* is unambiguously related to the pore size (see Figure 1b), knowing the *x* value, the effective pore size *D_eff_* can be deduced from Equation (1):

(2) $$D_{eff}=2\lambda \left(1+\frac{1+\sqrt{x+1}}{x}\right)$$

The division of the adsorption space into two parts is justified by the adsorption mechanism. The completion of the monolayer is usually easily identifiable on the adsorption isotherm as a change of the isotherm slope. The filling of the remaining space is indicated by the final plateau on the isotherm. This plateau occurs only if the size of the pores allows them to fill completely. Typically, such a mechanism is reflected by the type IV of the isotherm according to the IUPAC classification. Further considerations are limited to such cases as the complete pore filling by the adsorbed N_2_ and the type IV of its adsorption isotherm.

If we assume that the density of the adsorbate filling the pore is the same in each point of a cylindrical pore, the geometric ratio of the volumes in Equation (2) can be replaced by the ratio of adsorption capacities of the contact layer (*a*_0_) and the remaining pore space (*a_sec,s_*):

(3) $$x=\frac{a_{0}}{a_{sec,s}}$$

Both adsorption capacities can be deduced from the adsorption isotherm. The above notations, i.e., *a*_0_ and *a_sec,s_*, have been introduced to ensure compatibility with further mathematical considerations.

Unfortunately, most of the equations used to describe type IV isotherms have the dual-mode form. Such models are just the combination of two equations [37]. Examples of such approaches are Dubinin–Izotova adsorption isotherm (bimodal Dubinin–Astakhov equation) [38] or bimodal CMMS equation proposed by Rutherford [39]. These models treat independently both stages, i.e., the monolayer formation and the filling of the remained space. The adsorption isotherm equations are the sums of two independent parts. Hence, these and other similar approaches do not reflect the mechanism of the adsorption process. The molecules forming the multilayer are bound on the upper surface of the monolayer, i.e., on the molecules adsorbed in the contact layer. The quantity of fluid adsorbed in the multilayer should be dependent on adsorption in the monolayer. One of the few theoretical models able to generate the type IV isotherm and correctly reflect the mechanism of the process is the recently proposed fpv-GAB model [35]. As in the formalism of the original GAB [40,41,42] or Brunauer–Emmett–Teller (BET) [43] equations the fpv-GAB approach assumes the formation of vertical complexes of the adsorbed molecules, but their size is restricted. This restriction is equivalent to the finite volume of adsorption space and allows the generation of, among others, type IV isotherms, while the basic GAB equation can describe only type I, II, and III isotherms.

In the fpv-GAB approach the quantity of fluid adsorbed in the contact layer (on the primary adsorption sites) is described by Langmuir equation:

(4) $$a_{prim}=\frac{a_{0}Kh}{1+Kh}$$

where *a*_0_ is the concentration of primary centers equivalent to the monolayer capacity in the studied case, *K* is Langmuir constant reflecting the strength of solid–fluid interactions, and *h = p*/*p_s_* denotes the relative pressure, i.e., the fluid pressure in the gaseous phase (*p*) normalized with respect to the saturated vapor pressure *p_s_* at the given temperature. The formation of further layers is modeled using generalized Barton (GB) equation [35]:

(5) $$a_{sec}=c\left(a_{prim}+a_{sec}\right)\left(1-ka_{sec}^{n}\right)h$$

where *a_sec_* is the amount adsorbed in the multilayers next to the contact layer, i.e., on the secondary sites, *a_prim_* is the pressure-dependent adsorption amount in the monolayer calculated from Equation (4), *c* is a constant related to the energetics of adsorption in this space, i.e., fluid–fluid interactions, and *n* is the fitting parameter that ensures the best fit of experimental isotherm by Equation (5) in the high-pressure region. The constant *k* is related to the above-mentioned adsorption capacities of the remaining pore space (*a_sec,s_*), i.e., the adsorption amount in the multilayer at the saturation pressure. Since Equation (5) should lead to *a_sec_* = *a_sec,s_* for *h* = 1, the constant *k* can be expressed as [35]:

(6) $$k=\frac{c\left(a_{prim,s}+a_{sec,s}\right)-a_{sec,s}}{ca_{sec,s}^{n}\left(a_{prim,s}+a_{sec,s}\right)}$$

where *a_prim,s_* is the adsorption amount on primary sites for *h* = 1:

(7) $$a_{prim,s}=\frac{a_{0}K}{1+K}$$

The total amount adsorbed is the sum of monolayer and multilayer contributions:

(8) $$a=a_{prim}+a_{sec}$$

The fpv-GAB model is defined by the combination of Equations (4)–(8).

A shortcoming of the fpv-GAB approach can be the lack of lateral interactions between molecules adsorbed in the contact layer. This component of the interaction is particularly important when the adsorption occurs on homogeneous surfaces. Our previous studies [32,44,45] showed that the fit of the adsorption isotherms in the monolayer region for such systems is unsatisfactory without considering the effects of horizontal interactions in this layer. One of the simplest ways to include such interactions in the modeling of adsorption in the contact layers is to replace the Langmuir formula (Equation (4)) by Fowler–Guggenheim (FG) equation [46]:

(9) $$h=\frac{\Theta_{1}}{K\left(1-\Theta_{1}\right)}\exp\left[-A\Theta_{1}\right]$$

where *K* is the constant related to the strength of solid–fluid interactions, analogically to the same denoted constant in Equation (4), *A* is the parameter related to the strength of fluid–fluid lateral interactions, and Θ_1_ is the relative adsorption amount in the contact layer:

(10) $$\Theta_{1}=\frac{a_{prim}}{a_{0}}$$

The model, obtained by the modification of the original fpv-GAB approach (Equations (4)–(8)) and replacing Langmuir equation by the FG model, can be denoted as fpv-GAB-li, i.e., the fpv-GAB model with lateral interactions in the first layer. It is defined by the combination of Equations (4)–(6) and (8)–(10). Here, *a_prim,s_* in Equation (6) is determined from Equations (9) and (10) for *h* = 1. For *A* = 0, when the effects connected with lateral interactions are neglected, the fpv-GAB-li approach simplifies to the basic fpv-GAB one. It should be noted that the fpv-GAB-li approach has been proposed for the first time in the current work.

The mathematical formalism of both models, i.e., fpv-GAB and fpv-GAB-li, causes that calculation of the adsorption amount for the given value of relative pressure requires the use of numerical methods to determine the contribution from the multilayer (Equation (5)) in the case of both models and also the contribution from the monolayer in the case of fpv-GAB-li (Equations (9) and (10)). In the current study the bisection method has been used.

Appearing in Equation (3), the constants *a*_0_ and *a_sec,s_* as the best-fit parameters represent adsorption capacities in monolayer and multilayer. In the current study, the values calculated according to Equation (3) have been used to determine the pore diameter from Equation (2).

### 2.2. Monte Carlo Simulations of N_2_ Adsorption Isotherms inside CNTs

To verify the proposed methodology, the N_2_ adsorption has been simulated inside ideal, infinite, single walled carbon nanotubes (CNTs) of different effective diameters. We selected 30 zigzag type CNTs (see the first column of Tables S1 or S2 in the Supplementary Materials), with the diameters ranging from 1.06 nm to 12.10 nm. The diameter of the narrowest nanotube, *D_eff_* = 1.06 nm, is about three times greater than the monolayer thickness. This is a minimum pore size that enables us to observe two-step adsorption—first the formation of a monolayer close to the nanotube wall, followed by the filling of the remaining pore space. The adsorption isotherms have been simulated at *T* = 77.3 K (N_2_ boiling point) and relative pressure (*p*/*p_s_*) in the range 1.0 × 10^−8^–1.0 using hyper parallel tempering Monte Carlo (HPTMC) technique [47]. The values of the activity have been calculated as for an ideal gas. The cylindrical simulation boxes have consisted in fragments of CNT with the length of 4.23 nm. Periodic boundary conditions have been applied in the direction parallel to the tube axes. The formalism of the interaction energy calculations has been analogical to that described in [44]. The interaction parameters have been taken from [48,49].

### 2.3. Experimental Data

Nine experimental isotherms of N_2_ adsorption in cylindrical pores, measured at N_2_ boiling point, have been taken from the literature. They report the N_2_ adsorption inside carbon nanohorns (CNHs) [50] and in two series of MCM-41 materials [51,52]. Despite the fact that two parts, i.e., the conical and cylindrical ones, can be divided in the structure of CNHs [53], the internal pores of these materials are often modeled as tubular, i.e., CNHs are approximated by CNTs [54]. The presence of the conical part can be neglected because its volume is negligible. Previously, it was shown by us, via simulation, that there are no significant differences in the shapes of the low-temperature N_2_ isotherms inside infinite CNH and CNT of the same diameter, when the length of CNH is close to that observed for the real CNHs [55]. The MCM-41 type materials have cylindrical geometry of pores and a monomodal distribution of the pore diameters confirmed by XRD, TEM, and adsorption measurements [14,56]. The first series of the analyzed MCM-41 consists of three aluminosilicates samples modified with Al [51]. They were denoted according to the Si/Al molar ratios of the gels used for their preparation (60, 30, and 15) as Al-MCM-41(60), Al-MCM-41(30) and Al-MCM-41(15), respectively, although the final Si/Al ratios in the obtained samples are equal to 36.8, 19.9 and 12.2, respectively [51]. The second series consists of five pure-silica MCM-41 materials prepared using alkyltrimethylammonium halides with different length of alkyl group (C_12_, C_16_ and C_18_) [52]. The samples prepared using a surfactant containing hexadecyl group (C_16_) as a template were synthesized by three different methods [52]. Therefore, the samples in the second series have been denoted as MCM-41-12, MCM-41-16A, MCM-41-16B, MCM-41-16C and MCM-41-18. In all the experimental isotherms we have neglected the experimental points for the relative pressure above 0.9. The significant increase of the adsorption amount observed in this range results mainly from the adsorption in macropores and on the external surfaces of the real samples, not analyzed here.

### 2.4. Fitting of Adsorption Isotherms

The genetic algorithm proposed by Storn and Price [57] has been applied to fit simulated and experimental isotherms by the fpv-GAB (Equations (4)–(8)) and fpv-GAB-li (Equations (5), (6) and (8)–(10)) models. The best-fit parameters have been: *a*_0_, *K*, *c*, *n*, and *a_sec,s_* (fpv-GAB) and *a*_0_, *K*, *A*, *c*, *n*, and *a_sec,s_* (fpv-GAB-li). The fit quality has been evaluated using the determination coefficient (*DC*):

(11) $$DC=1-\frac{\sum_{i}{\left(a_{theo,i}-a_{sim/exp,i}\right)}^{2}}{\sum_{i}{\left(a_{sim/exp,i}-{\bar{a}}_{sim/exp}\right)}^{2}}$$

where *a_theo,i_* and *a_sim/exp,i_* are, respectively, the adsorbed amounts predicted by the model and the observed amounts (simulated or experimental) for at the *i*-th point of the isotherm, and *ā_sim/exp_* is the value of the simulated/experimental adsorption averaged over the whole isotherm. *DC* is equal to 1 for the perfect fit and decreases when the quality of the fit decreases.

## 3. Results and Discussion 

**Figure 2:**
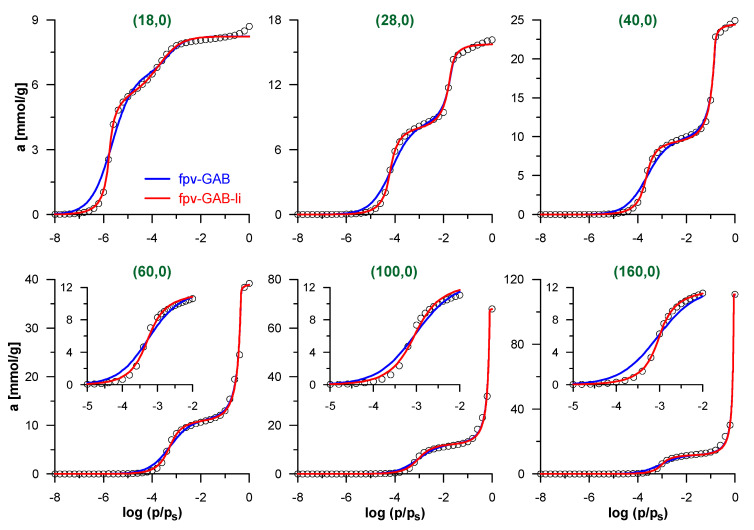
Simulated isotherms of N_2_ adsorption inside selected carbon nanotubes (CNTs) (points) and their fits by the models: fpv-GAB (Equations (4)–(8))—blue lines and fpv-GAB-li (Equations (5), (6) and (8)–(10))—red lines. Only selected simulated points are shown for clarity. The inserts in lower panels show the low-pressure part of isotherms, related to the formation of N_2_ monolayer on the pore wall.

We begin the discussion of the accuracy of the proposed methodology by the analysis of simulated isotherms of N_2_ adsorption inside CNTs, for which the effective pore diameters are perfectly known. Figure 2 shows selected simulated isotherms and their fits by fpv-GAB and fpv-GAB-li models. Tables S1 and S2 in Supplementary Materials present the obtained values of the best-fit parameters for all the model CNTs studied here. Both models allow good fit of the simulated data with the determination coefficient *DC* > 0.99. Quite systematic (close to monotonic) changes in the values of the best-fit parameters reflect the evolution of the shape of the isotherms as the diameter of the CNT increases. When the nanotubes become wider, the decreasing surface curvature causes the reduction of the effective energy of the solid–fluid interactions and the pressure related to monolayer formation increases. In consequence the values of K parameter in both models decrease, as well as the A value in the fpv-GAB-li approach. Similarly, the pressure of the filling of the central part of the pore also increases. The shape of the theoretical isotherm in this region is determined mainly by the values of the c and n parameters. The changes of both values are opposite, i.e., c decreases and n increases. It should be noted that the effects of both parameters can be formally compensating to some extent. The most important differences are observed in the region of the multilayer adsorption. Since the pore volume increases considerably as the diameter increases, the values of *a_sec,s_* also rise systematically. Surprisingly, the adsorption capacities of the monolayer (the *a*_0_ parameter) also increase. Such behavior was analyzed and explained by Salmas and Androutsopoulos [58]: due to geometric restrictions, the uptake of molecules on the highly curved cylindrical surface is much less efficient than on the flat surface. This effect reduces as the pore diameter increases.

The careful analysis of the theoretical curves in Figure 2 indicates that the fpv-GAB-li model fits the simulated isotherms better than fpv-GAB model (the values of *DC* are significantly higher). The main differences are observed in the low-pressure range, where the contact layer is formed. As the fpv-GAB-li model includes the lateral interactions between adsorbate molecules in this layer, it reproduces the steplike character of the monolayer formation better than the fpv-GAB model.

**Figure 3:**
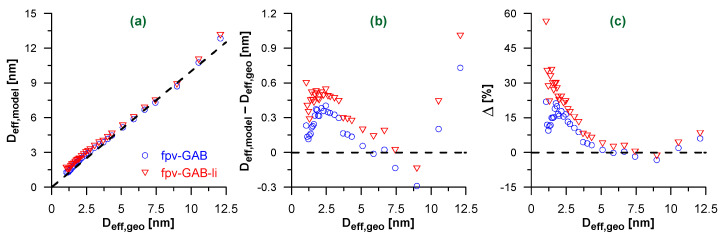
(**a**) Effective pore diameters *D*_*eff*,*model*_ calculated using Equation (2) and fpv-GAB (blue circles) or fpv-GAB-li (red triangles) fits of simulated isotherms of N_2_ adsorption in CNTs. The diameters are plotted as the function of the geometrical diameter of CNTs (*D*_*eff*,*geo*_). The dashed line represents *D*_*eff*,*model*_ = *D*_*eff*,*geo*_; (**b**,**c**) the absolute (**b**) and relative (**c**) differences between *D*_*eff*,*model*_ and *D*_*eff*,*geo*_. The horizontal dashed lines correspond to *D*_*eff*,*model*_ = *D*_*eff*,*geo*_.

Figure 3a shows the diameters of the model CNTs calculated using Equation (2) and the fitted monolayer and multilayer capacities (Tables S1 and S2 in Supplementary Materials). In general, both isotherm models lead to a realistic estimation of the pore diameters. The absolute errors (with the exception of the widest CNTs) do not exceed ca. 0.4 nm and 0.6 nm when using the best-fit parameters from the fpv-GAB and fpv-GAB-li models, respectively (Figure 3b).

The higher absolute errors for the widest nanotubes presumably result from imprecise estimation of the adsorption capacity in the multilayer. The isotherms for these systems contain only a few points in the final plateau. The relatively high errors for the narrow CNTs can be explained in two ways. On the one hand, the previously mentioned geometric restrictions [58] cause the noneffective packing of molecules in the contact layer. In such a case, the assumption of the uniform adsorbate density in the whole pore volume is not fulfilled and, according to Equation (2), the underestimation of the monolayer volume leads to the overestimation of the pore diameter. On the other hand, the density of the contact layer may slightly increase after its completion as the gas pressure approaches the saturation value [59,60,61,62]. The applied mathematical models do not account for such an effect. As is shown in Figure S1 in the Supplementary Materials, the monolayer capacity, i.e., the value of *a*_0_ parameter, corresponds with the adsorption amount at quite low value of relative pressure, while the shape of the isotherm suggests that some further increase in the adsorption amount for this layer may occur.

Surprisingly, although the fpv-GAB-li model fits better the adsorption isotherms, that is the fpv-GAB model that gives higher accuracy of the pore diameter determination. In fact, the evaluated contact layer capacity *a*_0_ is slightly lower in fpv-GAB-li model (Tables S1 and S2 in Supplementary Materials), which in turn leads to a slightly higher overestimation of the pore diameter than in fpv-GAB model. Nevertheless, the relative errors (Figure 3c) of *D_eff_* assessment are small and do not exceed 10 % for the nanotubes of diameter higher than ca. 3.5 nm. In the range 4.5–10 nm the errors are even less than 5%. Calculations of these quantities (Δ) include the division by the large geometric diameters. Thus, the relative errors are relatively small for the wider CNTs.

**Figure 4:**
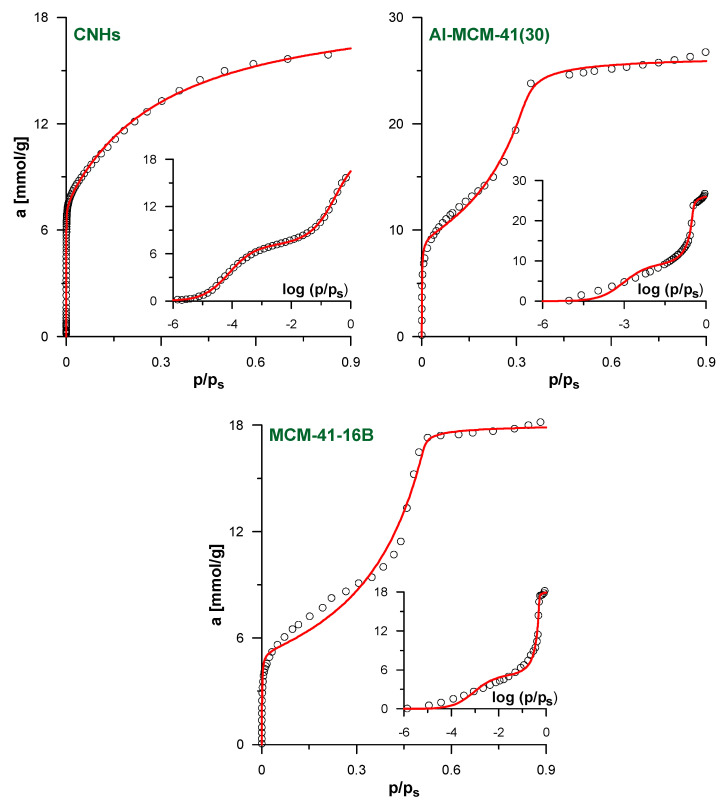
The results of the fitting of experimental N_2_ adsorption isotherms by fpv-GAB (Equations (4)–(8)) and fpv-GAB-li (Equations (4)–(6) and (8)–(10)) models for the samples CNHs, Al-MCM-41(30) and MCM-41-16B. The insets show the same data in logarithmic scale of the relative pressure. The points represent experimental data and lines reflect the predictions of the models. Since fpv-GAB-li equation is simplified to fpv-GAB (*A* ≈ 0) only one theoretical line is plotted for each system. In the case of CNHs and MCM-41-16B only selected experimental points are shown for clarity.

The verification of our model against the simulated isotherms justifies its application to analyze the experimental data. Figure 4 shows the results of the fitting of the experimental N_2_ adsorption isotherms for the selected studied systems. The results for the remaining systems and all fitting parameters are given in Supplementary Materials (Figures S2 and S3, Tables S3 and S4). As in all cases, the optimized value of *A* in fpv-GAB-li model is practically equal to 0; this approach simplifies to the basic fpv-GAB one (the values of the other best-fit parameters in both models become the same). As a consequence, only one theoretical curve is shown in each figure. We suggest that the apparent lack of the influence of lateral interaction on the adjustment of the experimental isotherms is a consequence of the heterogeneity of the pore walls in the real samples. If the energetic heterogeneity of the solid–fluid potential is large, the adsorption process begins on the sites of the highest energy, regardless the (weak) lateral fluid–fluid interactions. As the gas pressure increases, less adsorbing sites begin to be filled. The adsorbed amount increases progressively and the sharp step corresponding to the monolayer completion is not observed. Such behavior was observed for many the real adsorbents—see for example [32,44,45]. Nevertheless, the quality of the fits for the isotherms studied here is quite good, as indicated by the high *DC* values collected in Tables S3 and S4 in Supplementary Materials. The theoretical model reflects well the shape of the isotherms. The only exception is the medium-pressure range of the isotherms of the MCM-41-12 to MCM-41-18 materials. Here, the theoretical model does not precisely reproduce the experimental points at the beginning of the multilayer formation pressure range.

Table 1 gives the pore diameters calculated using Equation (2) and the adsorption capacities of the monolayer and the multilayer predicted by the applied models. For comparison, the diameters estimated using more sophisticated methods [32,50,51,52,63] are also shown. In general, the pore sizes calculated using our methodology are very similar to those reported in the literature. For example, in the case of CNHs, the estimated diameter agrees with the results of the GCMC simulations of Ohba et al. [50] and with the pore size estimated previously by us using the layer-by-layer NT-PFG adsorption isotherm model [32]. This justifies our statement that the proposed methodology is a simpler alternative to the previous complex NT-PFG model. As in [32], the calculated diameter is also significantly lower than the one estimated using the IDBdB method [63]. However, some results, such as observations with the use of high-resolution transmission electron microscopy [50], suggest that the real diameter is much lower in comparison to the predictions of the last method. In case of the MCM-41 materials, the diameters calculated with Equation (2) are in good agreement with those reported in the literature [51,52]. The differences do not exceed 0.3 nm. With the exception of the samples with the widest pores, i.e., MCM-41-16B and MCM-41-16C, the proposed approach overestimates the pore size similarly to in the case of the model CNTs—Figure 3. However, the trend of the diameter changes in both considered series of MCM-41 materials is reflected quite well. Therefore, the proposed methodology can be used as the simple tool for the rapid estimation of cylindrical pore diameters and their comparison between different samples.

## 4. Conclusions

We proposed a simple approach which allows the estimation of the diameter of cylindrical pores with the use of a N_2_ adsorption isotherm. It was based on the geometrical considerations and the division of adsorption space into two parts: the monolayer and the multilayer space. The ratio of the volumes of these two spaces is unambiguously related to the pore diameter. We have suggested to use the fpv-GAB adsorption isotherm model to fit the adsorption data. The ratio of the adsorption capacities in the monolayer and the multilayer obtained as the best-fit parameters is equivalent to the volume ratio. We have verified the proposed methodology with the use of the series of isotherms simulated inside CNTs and experimentally measured for different adsorbents. It has been proven that this approach not only describes adsorption isotherms well, but also it provides an estimation of the pore diameter that is comparable with the geometrical values or the results of the application of more sophisticated methods. With the exception of narrower pores (*D_eff_* < 3 nm), the relative error does not exceed a few percent. Moreover, our method reflects the trend of the changes in the sizes of the considered series of materials and can be used to compare different samples. Therefore, the fpv-GAB isotherm model can be treated as a simple tool which not only enables analysis of the shape of the isotherm theoretically, but also provides information about the pore diameter.

## Figures and Tables

**Table 1:** The comparison of effective pore diameters for all the studied samples calculated from Equation (2) and the best-fit parameters obtained from the fitting of N_2_ adsorption isotherms by theoretical models and the values obtained from more sophisticated methods published by others.

Sample	*D_eff_* [nm]		Method ^1^	References
	The Models (Current Work)	Literature Data		
CNHs	2.83	2.9 3.38 ± 0.48 2.96	GCMC IDBdB NT-PFG	[50] [63] [32]
Al-MCM-41(60)	3.22	3.10	BJH	[51]
Al-MCM-41(30)	3.56	3.54	BJH	[51]
Al-MCM-41(15)	3.74	3.56	BJH	[51]
MCM-41-12	3.37	3.07 3.08	BJH GM	[52]
MCM-41-16A	4.02	3.85 3.88	BJH GM	[52]
MCM-41-16B	4.47	4.66 4.55	BJH GM	[52]
MCM-41-16C	5.07	5.12 5.11	BJH GM	[52]
MCM-41-18	4.36	4.16 4.22	BJH GM	[52]

^1^ GCMC—the grand canonical Monte Carlo simulations, IDBdB—the improved Derjaguin–Broekhoff–de Boer method, NT-PFG—the nanotube polymolecular Fowler–Guggenheim adsorption isotherm, BJH—the Barrett–Joyner–Halenda method, GM—the geometrical considerations assuming hexagonal arrays of uniform pores of circular geometry.

## Data Availability

The data presented in this study are available on request from the corresponding author.

## Supplementary Materials

The following are available online at Table S1: The values of best-fit parameters obtained using fpv-GAB model for the description of all the simulated N_2_ adsorption isotherms inside CNTs; Table S2: As in Table S2 but for fpv-GAB-li model; Table S3: The values of best-fit parameters obtained using fpv-GAB model for the description of all the studied experimental N_2_ adsorption isotherms; Table S4: As in Table S3 but for fpv-GAB-li model; Figure S1: The comparison of the simulated N_2_ adsorption isotherm inside (28,0) CNT and its shape predicted by the fpv-GAB and fpv-GAB-li models and the contributions from the monolayer predicted by the models and the obtained values of adsorption capacities in this layer (the value of *a*_0_ parameter); Figure S2: The results of the fitting of experimental N_2_ adsorption isotherms by fpv-GAB and fpv-GAB-li models for the samples Al-MCM-41(60) and Al-MCM-41(15); Figure S3: As in Figure S2 but for samples MCM-41-12, MCM-41-16A, MCM-41-16C and MCM-41-18.
