# Supplementary material for: The Finite Pore Volume GAB Adsorption Isotherm Model as a Simple Tool to Estimate a Diameter of Cylindrical Nanopores
Source: Molecules. 2021 Mar 10;26(6):1509. doi: 10.3390/molecules26061509 (PMC8000439; doi:10.3390/molecules26061509)
Supplement: Supplementary file 1 [file molecules-26-01509-s001.pdf]

Supplementary Materials

for

The finite pore volume GAB adsorption isotherm model as a simple tool to estimate a diameter of cylindrical nanopores

Sylwester Furmaniak, Piotr A. Gauden, Maria Leżańska,
Radosław Miśkiewicz, Anna Błajet-Kosicka, Piotr Kowalczyk

number of pages 7
number of tables 4
number of figures 3

Table S1

The values of best-fit parameters obtained using fpv-GAB model (Equations (4)-(8)) for the description of all the simulated N_2 adsorption isotherms inside CNTs.

**Nanotube**	a_0 **[mmol/g]**	K	c	n	$a_{sec,s}$ **[mmol/g]**	DC
(18,0)	6.516	4.502×10^5	388.1	1.628	1.727	0.9896
(19,0)	7.340	1.955×10^5	322.6	0.3123	1.863	0.9915
(20,0)	7.308	1.237×10^5	189.5	0.8648	2.112	0.9910
(21,0)	7.461	7.961×10^4	97.17	0.9544	2.671	0.9907
(22,0)	7.867	5.062×10^4	58.63	1.612	3.285	0.9939
(23,0)	7.900	3.827×10^4	60.61	2.365	3.990	0.9932
(24,0)	7.960	2.997×10^4	49.37	3.664	4.489	0.9932
(25,0)	8.096	2.370×10^4	34.62	4.688	5.080	0.9945
(26,0)	8.251	1.908×10^4	25.68	3.445	6.029	0.9933
(27,0)	8.355	1.595×10^4	20.38	5.515	6.859	0.9946
(28,0)	8.421	1.373×10^4	17.90	8.662	7.328	0.9940
(29,0)	8.557	1.179×10^4	14.64	9.315	7.618	0.9934
(30,0)	8.740	1.013×10^4	11.30	8.181	8.237	0.9942
(32,0)	8.942	8.072×10^3	8.396	10.66	9.809	0.9939
(34,0)	9.160	6.622×10^3	6.502	12.88	10.88	0.9942
(36,0)	9.373	5.569×10^3	5.178	13.24	12.45	0.9949
(38,0)	9.556	4.819×10^3	4.335	19.42	13.36	0.9948
(40,0)	9.759	4.204×10^3	3.652	20.17	14.66	0.9954
(44,0)	10.11	3.351×10^3	2.823	23.65	17.30	0.9959
(48,0)	10.38	2.826×10^3	2.375	29.55	19.84	0.9955
(52,0)	10.90	2.268×10^3	1.795	46.88	22.20	0.9961
(56,0)	11.07	2.041×10^3	1.654	50.87	24.91	0.9954
(60,0)	11.25	1.850×10^3	1.522	57.03	27.64	0.9950
(70,0)	11.67	1.509×10^3	1.289	78.75	34.45	0.9942
(80,0)	11.99	1.299×10^3	1.162	82.90	41.44	0.9947
(90,0)	12.03	1.231×10^3	1.110	115.5	48.54	0.9922
(100,0)	12.38	1.084×10^3	1.018	175.7	55.42	0.9932
(120,0)	12.74	938.0	0.9433	166.2	69.74	0.9946
(140,0)	12.17	1.039×10^3	0.9736	159.0	84.38	0.9882
(160,0)	11.76	1.115×10^3	0.9949	153.4	99.01	0.9845

Table S2

As in Table S2 but for fpv-GAB-li model (Equations (5)-(6) and (8)-(10)).

**Nanotube**	a_0 **[mmol/g]**	K	A	c	n	$a_{sec,s}$ **[mmol/g]**	DC
(18,0)	5.493	1.414×10^5	2.861	1.288×10^4	0.1322	2.742	0.9982
(19,0)	6.351	8.000×10^4	2.428	1.838×10^3	0.4185	2.671	0.9962
(20,0)	6.428	4.502×10^4	2.551	1.066×10^3	0.6924	2.808	0.9979
(21,0)	6.866	2.979×10^4	2.386	281.3	0.8024	3.066	0.9974
(22,0)	6.917	1.963×10^4	2.518	186.5	0.9117	4.208	0.9991
(23,0)	6.980	1.387×10^4	2.600	146.1	1.285	4.928	0.9984
(24,0)	7.255	1.058×10^4	2.538	75.55	2.899	5.201	0.9983
(25,0)	7.516	8.371×10^3	2.471	46.26	3.993	5.667	0.9993
(26,0)	7.709	6.738×10^3	2.443	33.65	2.903	6.581	0.9976
(27,0)	7.897	5.549×10^3	2.421	23.98	5.093	7.319	0.9987
(28,0)	7.999	4.620×10^3	2.452	20.16	8.462	7.744	0.9980
(29,0)	8.115	3.966×10^3	2.441	16.33	9.285	8.022	0.9975
(30,0)	8.333	3.489×10^3	2.393	12.57	7.983	8.637	0.9980
(32,0)	8.561	2.734×10^3	2.394	9.095	10.55	10.18	0.9974
(34,0)	8.784	2.243×10^3	2.376	6.969	12.77	11.25	0.9976
(36,0)	9.000	1.903×10^3	2.347	5.500	13.10	12.82	0.9981
(38,0)	9.186	1.654×10^3	2.325	4.574	19.03	13.73	0.9978
(40,0)	9.389	1.474×10^3	2.274	3.833	20.12	15.02	0.9982
(44,0)	9.749	1.234×10^3	2.160	2.934	24.28	17.65	0.9983
(48,0)	10.02	1.061×10^3	2.098	2.454	30.56	20.17	0.9977
(52,0)	10.57	1.038×10^3	1.699	1.837	51.89	22.49	0.9975
(56,0)	10.75	931.1	1.684	1.688	55.65	25.20	0.9967
(60,0)	10.94	855.3	1.641	1.550	62.58	27.92	0.9962
(70,0)	11.38	738.7	1.504	1.305	87.11	34.71	0.9951
(80,0)	11.71	667.5	1.407	1.175	80.27	41.72	0.9955
(90,0)	11.76	580.1	1.550	1.119	126.2	48.79	0.9931
(100,0)	12.14	581.0	1.307	1.023	175.5	55.66	0.9938
(120,0)	12.54	558.6	1.099	0.9465	166.0	69.99	0.9950
(140,0)	11.92	407.0	1.845	0.9771	158.9	84.66	0.9893
(160,0)	11.52	340.6	2.232	0.9977	153.4	99.24	0.9859

Table S3

The values of best-fir parameters obtained using fpv-GAB model (Equations (4)-(8)) for the description of all the studied experimental N_2 adsorption isotherms.

**Sample**	$\boldsymbol{a_0}$ **[mmol/g]**	$\boldsymbol{K}$	$\boldsymbol{c}$	$\boldsymbol{n}$	$\boldsymbol{a_{sec,s}}$ **[mmol/g]**	***DC***
CNHs	7.145	1.261×10^{4}	131.5	1.732×10^{-2}	9.332	0.9992
Al-MCM-41(60)	8.544	1.311×10^{3}	2.169	5.733	13.48	0.9936
Al-MCM-41(30)	9.201	1.068×10^{3}	1.793	17.20	16.76	0.9939
Al-MCM-41(15)	8.189	1.018×10^{3}	1.701	6.366	15.97	0.9934
MCM-41-12	6.283	2.499×10^{3}	2.463	19.21	10.61	0.9913
MCM-41-16A	6.768	1.877×10^{3}	1.701	30.82	14.54	0.9898
MCM-41-16B	5.169	1.116×10^{3}	1.349	55.97	12.74	0.9906
MCM-41-16C	5.724	1.132×10^{3}	1.300	61.97	16.53	0.9864
MCM-41-18	6.545	1.380×10^{3}	1.563	31.83	15.64	0.9873

Table S4

As in Table S3 but for fpv-GAB-li model (Equations (5)-(6) and (8)-(10)).

**Sample**	$\boldsymbol{a_0}$ **[mmol/g]**	$\boldsymbol{K}$	$\boldsymbol{A}$	$\boldsymbol{c}$	$\boldsymbol{n}$	$\boldsymbol{a_{sec,s}}$ **[mmol/g]**	***DC***
CNHs	7.145	1.261×10^{4}	~0	131.5	1.732×10^{-2}	9.332	0.9992
Al-MCM-41(60)	8.544	1.311×10^{3}	~0	2.169	5.733	13.48	0.9936
Al-MCM-41(30)	9.201	1.068×10^{3}	~0	1.793	17.20	16.76	0.9939
Al-MCM-41(15)	8.189	1.018×10^{3}	~0	1.701	6.366	15.97	0.9934
MCM-41-12	6.283	2.499×10^{3}	~0	2.463	19.21	10.61	0.9913
MCM-41-16A	6.768	1.877×10^{3}	~0	1.701	30.82	14.54	0.9898
MCM-41-16B	5.169	1.116×10^{3}	~0	1.349	55.97	12.74	0.9906
MCM-41-16C	5.724	1.132×10^{3}	~0	1.300	61.97	16.53	0.9864
MCM-41-18	6.545	1.380×10^{3}	~0	1.563	31.83	15.64	0.9873

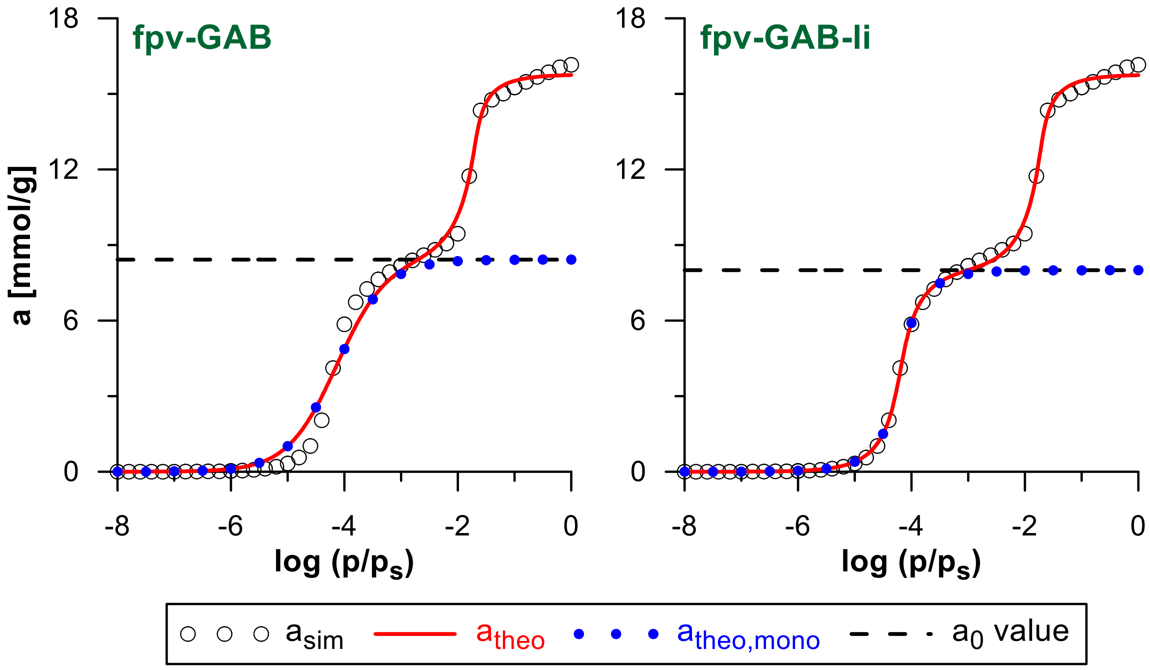

Figure S1. The comparison the simulated N_2 adsorption isotherm inside (28.0) CNT – open circles (selected point are shown) and its shape predicted by the fpv-GAB (Equations (4)-(8)) and fpv-GAB-li (Equations (5)-(6) and (8)-(10)) models – red lines. The contributions from the monolayer predicted by the models are also shown as blue full circles. In addition, the vertical dashed lines represent the obtained values of adsorption capacities in this layer (the value of a_0 parameter) – see Tables S1 and S2.

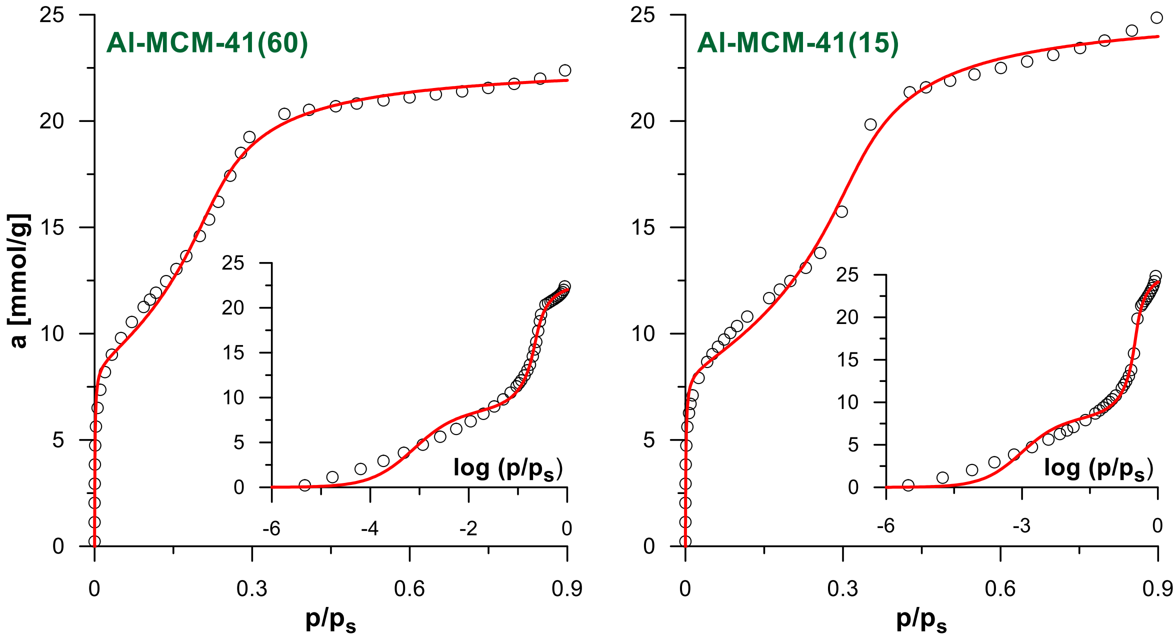

Figure S2. The results of the fitting of experimental N_2 adsorption isotherms by fpv-GAB (Equations (4)-(8)) and fpv-GAB-li (Equations (5)-(6) and (8)-(10)) models for the samples Al-MCM-41(60) and Al-MCM-41(15). The insets show the same data in logarithmic scale of the relative pressure. The points represent experimental data and lines reflect the predictions of the models. Since fpv-GAB-li equation is simplified to fpv-GAB ($A \approx 0$) only one theoretical line is plotted for each system.

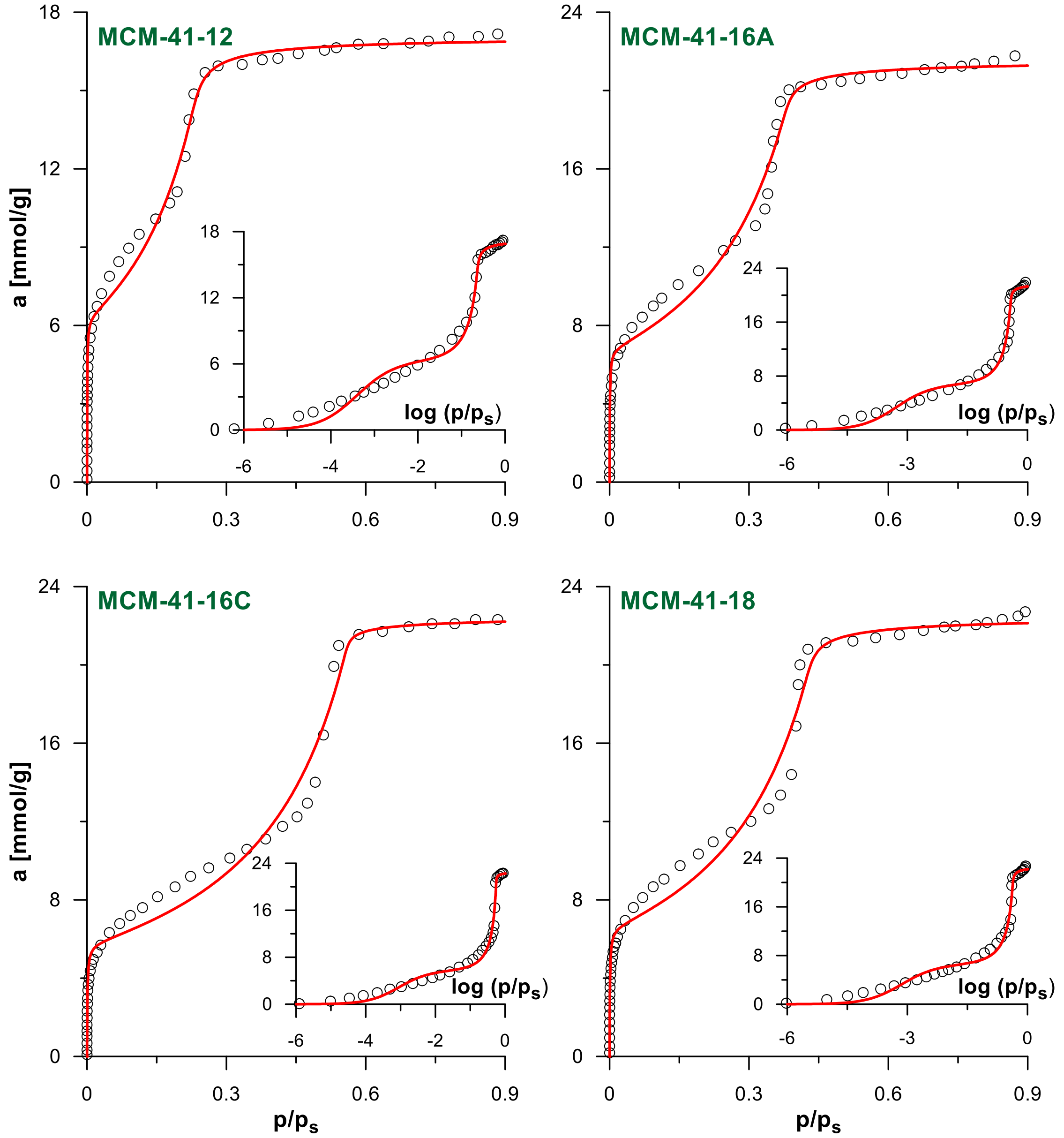

Figure S3. As in Figure S2 but for samples MCM-41-12, MCM-41-16A, MCM-41-16C and MCM-41-18. Only selected experimental points are shown for clarity.
